# Evaluation and phenotypic plasticity of taro [*Colocasia esculenta* (l.) Schott.] genotypes for nutrient and anti-nutrient composition

**DOI:** 10.1371/journal.pone.0291358

**Published:** 2023-09-13

**Authors:** Esther Fobi Donkor, Daniel Nyadanu, Richard Akromah, Kingsley Osei, Daniel Asomaning Odoom

**Affiliations:** 1 Department of Horticulture and Crop Production, School of Agriculture, University of Energy and Natural Resources, Sunyani, Ghana; 2 Plant Breeding Division, Cocoa Research Institute, Accra, Tafo Ghana; 3 Department of Crop and Soil Sciences, Kwame Nkrumah University of Science and Technology, Kumasi, Ghana; 4 Pathology Department, Crop Research Institute- Fumesua, Council for Scientific and Industrial Research, Ejisu, Ghana; KGUT: Graduate University of Advanced Technology, ISLAMIC REPUBLIC OF IRAN

## Abstract

The study was carried out to determine the nutritional and anti-nutritional composition of taro genotypes and also determine the phenotypic plasticity of the genotypes in two agro ecological zones in Ghana. The towns and zones were Bunso in the semi deciduous forest (an upland) and Tano Dumasi in the forest savannah transition agro-ecological (a waterlogged area) zone in the Eastern and Ashanti regions respectively.Two (2) freshly harvested corms of each genotype from each location were assessed for their nutritional (moisture, protein, carbohydrate, ash and fat) and anti-nutritional (phytate, oxalate and tannin) composition Data collected were subjected to analysis of variance and AMMI analysis using GenStat 12 edition to assess the effect of genotype, environment and their interaction on the traits studied. Phenotypic plasticity for the genotypes and the traits studied was also calculated. Pearson correlation was also conducted to assess the relationship between the traits studied. There were significant differences among the genotypes for nutrient and anti-nutrient composition except for percentage fat, indicating enough genetic variability among the genotypes, giving room for good selection progress for development of taro varieties. A higher magnitude of the environment over genotype and genotype by environment interaction observed indicates the influence of environment in the expression of the nutritional and anti-nutritional traits. Observed varied phenotypic plasticity among the genotypes for the nutrient and anti-nutrients composition also indicates varied adaptation of the genotypes to the environment. Genotypes BL/SM/115, CE/MAL/32 and CE/IND/16 and hybrids KAO19 × CE/MAL/32 and CE/IND/16×KAO19, CE/IND/16 × BL/SM/10, and CE/IND/16 × BL/SM/115 which recorded high nutrients and low anti-nutrients content and were stable across the environments can be released to farmers for cultivation. They could also be included in breeding programs for the development of enhanced nutritional quality of taro in Ghana.

## 1.0 Introduction

Root crops such as taro [*Colocasia esculenta* (l.) Schott.] play a major role in the food security of many developing countries as it serves as an important food or subsistence crop for millions of people in these developing countries [1] (Misra *et al*., 2008). Taro is ranked the fifth most utilized root tuber after cassava, potato, sweet potato and yam in the world [2, 3] (Bamidele *et al*., 2014; Igbabul *et al*., 2014).It also has the potential of improving the livelihood of many poor people who depend on it for food [4] (Akalu and Geleta, 2017).

Taro has a lot of food and medicinal properties.All the plant parts that is the young leaves (petiole and blade), corms and cormels are used as vegetables in sauces and soups [5–7] (Owuamanam *et al*., 2010; Angami *et al*., 2015; Matthews, 2014**).** The corms can be consumed after baking, roasting, steaming or boiling. It can also be fried as chips, dried and made into flour for preparation of pastries [8] (Krishnapriya and Suganthi, 2017). The corms contain anthocyanins, cyanidin 3-glucoside and flavonoids which improve blood circulation by strengthening the capillaries of the heart [9] (Wagner, 1985). It also acts as potent antioxidants and anti-inflammatory agent and inhibit human cancer cell growth [10] (Youdim *et al*., 2000).

Taro has the potential of minimizing malnutrition among poor rural population since it is rich in mineral and micro nutrients [6] (Angami *et al*., 2015*)*. The corms are good source of starch (70–80 g/100 g dry taro), fiber (0.8%), and ash (1.2%) [11] (Soudy *et al*., 2010).It is also rich in thiamine, riboflavin, iron, phosphorus, and zinc and a very good source of vitamin B6, vitamin C, niacin, potassium, copper, and manganese but low in protein (1.5%) and fat (0.2%) [12] (Quach *et al*., 2001). The starch of taro corms is hypoallergenic, gluten free and has smaller granule size which makes it easy to digest [13, 14] (Jane *et al*., 1992; Deepika et al., 2020).Anti-nutritional compounds reduce the nutrient utilization and food intake of most corms [4] (Akalu and Geleta, 2017). Corms including taro have traces of anti-nutritional components such as cyanoglucosides, oxalates, phytic acid, tannins, phenolics and protease inhibitors which when consumed may have adverse effects on health through inhibition of protein digestion, growth, and Fe and Zn absorption [15, 16] (Mitharwal *et al*., 2022; Gemede and Ratta, 2014).

The oxalate compounds in raw taro are responsible for theacridity in taro therefore, it is advisable to process taro before consumption [17] (Bradbury *et al*., 1995). In Ghana, taro varieties with higher nutritional content and low oxalate, phytate and tannin content are unavailable. There is also limited genotype by environment interaction (GEI) analysis on nutritional components of taro limiting selection and recommendation of suitable genotypes which possess desirable nutritional traits for food, feed and industrial use [18, 19] (Andrade *et al*., 2016; Tumwegamire *et al*., 2016). Breeding for improved nutrient content in taro will go a long way to improve the nutritional status of most rural poor in Ghana. Nutritional composition of crops is highly influenced by genotype by environment interaction and therefore the genotypes generated should be evaluated under different environmental conditions to assess their phenotypic plasticity of environment. Phenotypic plasticity of a genotype is the extent to which their performance varies under different environmental conditions [20, 21] (Bradshaw, 2006; Lande, 2009).Phenotypic plasticity evaluations are also important in the face of climatic change [22] (Hidayatullah *et al*., 2020). When the plasticity of a genotype for a particular trait near zero, the genotype is deemed stable and when it is far from zero, the genotype is plastic that is unstable [23] (Tripodi *et al*., 2020). Understanding the phenotypic plasticity of genotypes is crucial in breeding programs as it aids in selecting genotypes for varietal improvement and for predicting theirperformances in different environments [24] (Tchokponhoue *et al*., 2019). Phenotypic plasticity helps the breeder to understand the crops adaptation and the links between phenotype and genotype [25, 26] (Sadras and Trentacoste, 2011; Mohammadi, 2014). The objective of the study therefore was to evaluate and determine the phenotypic plasticity of parents and single cross hybrids of taro for nutritional and anti-nutritional composition under two agro-ecological environments and select stable genotypes of taro that are low in anti-nutrients and rich in beneficial nutrients and health related functional traits for varietal development of taro in Ghana. Recently, [51] Ouédraogo et al (2023) have reported on the nutritional composition in Bukina Faso, [33] Boampong et al (2019) also reported on nutritional composition of taro accessions in Ghana, however this paper assesses the nutritional and anti-nutritional composition of parents and hybrids of taro in two contrasting environments.

## 2.0 Materials and methods

### 2.1 Research materials

Harvested corms of equal weight from parents and single cross hybrids were used in the chemical analysis.The genotypes used for the chemical analysis were involved in the evaluation of yield and yield components of taro. Table 1 shows the names of parents, hybrids and details of crosses used in the research. The genotypes used as parents except for KAO 19 which is a local accession from Ghana were introduced in-vitro into Ghana as part of the germplasm distributed during 2004–2014 by Centre for Pacific Crops and Trees (CePaCT) as part of their “Fighting TLB Disease in Samoa and at Global Level Through Networking and Sharing Genetic Resources” program due to the narrow genetic base of taro in Ghana. All the exotic genotypes were conserved at Plant Genetic Resource Research Institute (PGRRI) of the Council for Scientific and Industrial Research (CSIR), Bunso in the Eastern region of Ghana.

**Table 1:** Names of parents, crosses and details of crosses used in the research.

Genotypes	Details	Name
GEN1	P1	KAO19
GEN2	P2	BL/SM/10
GEN3	P3	BL/SM/115
GEN4	P4	CE/MAL/32
GEN5	P5	CE/IND/16
GEN6	P1 × P2	KAO19 × BL/SM/10
GEN7	P1 × P3	KAO19 × BL/SM/115
GEN8	P1 × P4	KAO19 × CE/MAL/32
GEN9	P1 × P5	KAO19 × CE/IND/16
GEN10	P2 × P1	BL/SM/10 × KAO19
GEN11	P2 × P3	BL/SM/10 × BL/SM/115
GEN12	P2 × P4	BL/SM/10 × CE/MAL/32
GEN13	P2 × P5	BL/SM/10 × CE/IND/16
GEN14	P3 × P1	BL/SM/115 × KAO19
GEN15	P3 × P2	BL/SM/115 × BL/SM/10
GEN16	P3 ×P4	BL/SM/115 × CE/MAL/32
GEN17	P3 × P5	BL/SM/115 × CE/IND/16
GEN18	P4 × P1	CE/MAL/32 × KAO19
GEN19	P4 × P2	CE/MAL/32 × BL/SM/10
GEN20	P4 × P3	CE/MAL/32 × BL/SM/115
GEN21	P4 × P5	CE/MAL/32 × CE/IND/16
GEN22	P5 × P1	CE/IND/16 × KAO19
GEN23	P5 × P2	CE/IND/16 × BL/SM/10
GEN24	P5 × P3	CE/IND/16 × BL/SM/115
GEN25	P5 × P4	CE/IND/16 × CE/MAL/32

CE = Colocasia esculenta, BL = Breeding Line, MAL = Malaysia, SM = Samoa and IND = Indonesia.

### 2.2 Research sites

#### 2.2.1 Environment 1 –Bunso

The research was conducted at Plant Genetic Resources Research Institute of the Council for Scientific and Industrial Research (PGRRI–CSIR), research field at Bunso in the Eastern region of Ghana. Bunso is located in the semi deciduous forest agro-ecological zone at longitude 0°27.634´W, latitude 6°17.715´N and altitude 208.4m. The region is known for the large-scale cultivation and consumption of taro.The Bunso research site was an upland.

#### 2.2.2 Environment 2 –Tano Dumasi

The research was also conducted at the Ministry of Fisheries and Aqua Culture research site at Tano Dumasi in the Sekyere South District of Ashanti Region. Tano Dumasi lies in the forest savannah transition agro-ecological zone at longitude1°30.2360´W, latitude 6°53.2530´N and altitude 277.1m. Tano Dumasi was previously known for the cultivation of taro but currently most of the taro fields are used for rice cultivation due to the incidence of taro leaf blight disease. The field was a waterlogged area or a lowland.

The two fields were selected for the research to also assess the effect of moisture on the nutrient and anti-nutrient composition of taro, since taro is believed to be cultivated mostly in waterlogged areas in Ghana.

Table 2 shows the monthly average temperature and rainfall at the research sites for the period of the research. Table 3 shows the routine soil analysis and the soil type of the research sites.

**Table 2:** Monthly average for temperature (°C) and rainfall(mm) from April 2019 to December 2019 at two research sites.

Month	Bunso		Tano Dumasi	
	Temp (°C)	Rainfall (mm)	Temp (°C)	Rainfall (mm)
April	27.5	19.4	22.4	129.3
May	27.2	21.7	22.2	174.4
June	26.5	20.4	21.6	214.3
July	25.2	12.8	21.2	157.5
August	25.3	9.4	21.0	89.9
September	26.1	20.2	21.1	165.2
October	26.0	27.5	21.5	153.3
November	26.6	18.2	21.7	74.3
December	25.3	4.2	20.8	25.8
Mean	26.2	17.1	21.5	131.6

Source: Cocoa Research Institute of Ghana (CRIG) sub-station, Bunso, 2020

www.ghanameteo.gov.gh.gmet. Assessed in March 2020.

**Table 3:** Routine soil analysis and soil type of Bunso and Tano Dumasi.

ENVIRON.	pH	AVA. P mg/kg	% TOT N	Exch. Bases (cmol/kg)				Exch. Acidity		% Org. Carbo	% Org. Mat.	Soil type
				K	Ca	Mg	Na	Al	H			
**Bunso**	5.74	6.36	0.23	0.36	3.40	1.60	0.04	0.35	0.16	1.39	2.41	Sandy Loam
**Tano Dumasi**	5.85	5.58	0.26	0.33	6.80	2.00	0.04	0.38	0.19	1.72	2.96	Clay Loam

P = Phosphorus; K = Potassium; Ca = Calcium; Mg = Magnesium; Na = Sodium; Al = Aluminum; N = Nitrogen

### 2.3 Nutrient and anti-nutrient analysis

Two corms were randomly selected from the harvested corms from each genotype and sent to the Food Science Laboratory of the Food Science and Technology Department of Kwame Nkrumah University of Science and Technology (KNUST) [27]. The AOAC (1990) protocol for chemical analysis was used for nutrient and anti-nutrient analysis. The analysis was done in triplicates for all the nutritional and anti-nutritional traits.

#### 2.3.1 Protein determination

The protein content was determined using the Kejdahl method as follows:

*Digestion*. Two (2) g of sample and a half of selenium–based catalyst tablets were put in a digestion flask with a few anti-bumping agents. Twenty-five (25) ml of concentrated H_2_SO_4_ was then added and shaken until the entire sample was thoroughly wet. The flask was put on digestion burner and heated slowly until boiling ceased and the resulting solution was clear. The mixture was then cooled to room temperature. The digested sample solution was transferred into a 100 ml volumetric flask and made up to the mark.

*Distillation*. Twenty- five (25) ml of 2% boric acid was pipetted into a 250 ml conical flask and two drops of mixed indicator added. The conical flask and its contents were placed under a condenser in such a position that the tip of the condenser is completely immersed in solution. Ten (10) ml of the digested sample solution was measured into the decomposition flask of the Kejdahl unit and fixed. Excess of 40% NaOH (about 15–20 ml) was added to it.The ammonia produced was distilled into the collection flask with the condenser tip immersed in the receiving flask till a volume of about 150 ml– 200 ml was collected.

*Titration*. The distillate was titrated with 0.1N HCl solution. Acid was added until the solution was colorless. The nitrogen content was determined in triplicate, and a blank determination was run using the same amount of all reagents as used for the sample.

The total nitrogen was then calculated as follows:

$$Total\;nitrogen=\frac{50\times (Va-Vb)\times NA\times 14.01\times 100}{W\times 10\times 100}$$ (1)

Va- volume in ml of standard acid used in titration

Vb- volume in ml of standard acid used in blank

NA- normality of acid

W- Weight of sample taken

#### 2.3.2 Ash content

Five (5) g sample was weighed into a tarred crucible. The crucible was placed in a cool muffle furnace and ignited for 2 hours at about 600 degrees. The muffle furnace was turned off and opened until the temperature dropped to at least 250 degrees preferably lower. The door was opened carefully to avoid losing ash that may be fluffy. Safety tongs were used to quickly transfer crucibles to a desiccator with a porcelain plate and desiccant. The desiccator was closed and crucibles allowed to cool prior to weighing. The ash content was calculated as follows:

$$\%\;ASH=\frac{wt\;of\;ash}{wt\;of\;sample}\times 100$$ (2)

#### 2.3.3 Fat content

The fat content was extracted using the soxhlet extraction as follows:

A previously dried (air oven at 100°C) sample was accurately weighed into 250 ml round bottom flask. Five (5) grams of dried sample was weighed unto a 22 ×80mm paper thimble or a folded filter paper. A bit of cotton or glass wool was placed into the thimble to prevent loss of the sample. One hundred and fifty (150) ml of petroleum spirit B.P 40–60°C was added to the round bottom flask and the apparatus assembled. Condenser was connected to the soxhlet extractor and reflux for 4–6 hours on the heating mantle. After extraction, the thimble was removed and the solvent recovered by distillation. The flask and fat/oil were heated in an oven at about 103°C to evaporate the solvent. The flask and its content were cooled to room temperature in a desiccator.

The flask was weighed and weight of fat/oil collected determined as follows:

$$\%Fat=\frac{fat\;collected\;}{weight\;of\;sample}\times 100$$ (3)

#### 2.3.4 Moisture content

The moisture content was analyzed using the oven drying method as follows:

Five (5) g of sample was transferred to previously dried and weighed dish. The dish was placed in an oven thermostatically controlled at 105 degrees for 5 hours. The dish was removed and placed in a desiccator to cool to room temperature and weighed. The dish was dried again for 30 minutes, cooled down and weighed. The drying was repeated, cooled and weighed until a constant weight was reached. The moisture content was determined as follows:

$$\%\;moisture\;(wt/wt)=\frac{wt\;of\;water\;in\;sample}{wt\;of\;wet\;sample}\times 100$$ (4)

#### 2.3.5 Carbohydrate

The total carbohydrate was calculated as follows

%carbohydrate=100−(%moisture+%Fat+%protein+%Ash) (5)

#### 2.3.6 Phytate determination

Four (4) g of taro sample was soaked in 100ml of 2% HCl for 3 hours and filtered through Whatman filter paper. Twenty-five (25) ml of the filtrate was weighed into 250 ml conical flask and 5ml of 0.3% ammonium thiocyanate solution added as an indicator and 53.5ml distilled water was added to give the desired acidity. The solution was titrated with standard iron III chloride solution which contained about 0.001905 g of iron per ml until a brownish yellow colour was attained and persisted for 5 minutes. The phytate content was calculated as:

$$Phytate\;(mg/100g)=\frac{titre-blank\;titre)\times titrant\times molar\;mass\;of\;phytic\;acid\times 100}{wt\;of\;sample}$$ (6)

#### 2.3.7 Oxalate determination

The oxalate content in all the samples were analyzed by following titration method using KMnO_4_ described in [27] AOAC (1990). In the determination of oxalate, 1 g of each selected samples were weighed and mixed with 20 ml of 0.1M HCl in a 50ml beaker to extract total oxalate. All beakers with samples and extracting solvents were kept in a water bath at 100°C for 30 minutes, later filtrated using Wattman No 1 filter paper and 0.5 ml of 5% calcium chloride was added to the filtrate to precipitate out calcium oxalate, the precipitate was separated by centrifugation at 3500 rpm for 15 minutes, and supernatant was discarded. The calcium oxalate precipitate was washed with 2 ml of 0.35 M ammonium hydroxide and then dissolved in 0.5 M of sulphuric acid. The dissolved solution was titrated with 0.1 M of potassium permanganate at 60°C till faint pink color persisted for at least 15 seconds. The oxalate content was calculated by using stoichiometric formula. The total oxalate contentwas expressed in mg/100 g of dry weight.


$$Oxalate=\frac{titre\times titrant\times molar\;mass\;of\;oxalic\;acid\times dilution\times 100}{wt\;of\;sample}$$ (7)

#### 2.3.8 Tannin determination

0.2 g of the taro sample was soaked in 10 ml of 70% acetone and then placed in an ice bath (to prevent acetone from evaporation). The set up was shaken for 12–15 minutes to extract tannin.The solution was allowed to cool for about 30 minutes and then filtered to collect the supernatant. Then 0.5 ml of the supernatant was placed in a test tube and 0.5 ml of distilled water was added followed by 0.5 ml of Folins’ reagent and 2.5 ml of 20% Na_2_CO_3_ solution. The test tube was vortexed and incubated at room temperature for 40 minutes. The absorbance of the resulting solution was read at 725 nm with a calorimeter and standard tannic acid curve plotted. Concentration of the sample was extrapolated from the plot.

No permit was required for the research because it did not involve any animal life and the Lab protocols used were referenced.

## 3.0 Statistical analysis

The Analysis of Variance (ANOVA) was conducted for the nutrient and anti-nutrient composition using GenStat 12^th^ edition [28] (GenStat, 2009) to determine the significance of the genotypes across the environment. The means were separated using the LSD at 5% significant level.

AMMI analysis was conducted using GenStat 12 edition to assess the effect of genotype, environment and their interaction on the nutrient and anti-nutrient compositions.

The coefficient of relative phenotypic plasticity (CRP) for the genotypes used and each trait was calculated according to [29] Dingemanse *et al*. (2010) as follows:

$$CRP=\frac{Vi}{VPp}$$ (8)

Where Vi is the variance of the individual (*i*)

VPp is the overall phenotypic variance of the population.

Genotypic means were used to compute Pearson correlation coefficients between the nutrient and anti-nutrient composition across the environments using Statistix version 9.1 [30] (Statistix, 2013).

## 4.0 Results

### 4.1 Diversity among the genotypes for the nutrient and anti-nutrient compositions

The combined means of the parents and single cross hybrids for the nutrient and anti-nutrient compositions across the environment are presented in Table 4 and in the various environments as S1 and S2 Tables in S1 File. The analysis of variance (ANOVA) revealed highly significant (p<0.001) differences among the genotypes for the nutrient and anti-nutrient composition except for percentage fat content (Table 4).

**Table 4:** Combined means of taro genotypes for nutrient and anti-nutrient composition across two (2) environments.

GEN	PT	OX	TN	MC	ASH	PTT	FAT	TC
KAO19	268.48	1409.21	838.84	67.91	2.7	8.25	0.94	79.65
BL/SM/10	74.13	1701.47	887.80	64.08	1.89	10.28	1.86	79.33
BL/SM/115	65.26	563.58	314.42	53.97	3.87	14.65	0.84	75.38
CE/MAL/32	37.16	566.73	301.94	57.86	3.84	11.93	0.75	79.81
CE/IND/16	108.05	1347.63	727.84	62.48	3.01	11.73	0.77	82.37
KAO19 × BL/SM/10	199.59	727.26	463.43	57.97	2.87	7.79	0.95	76.59
KAO19 × BL/SM/115	173.06	372.89	272.98	60.88	3.7	12.17	0.89	76.38
KAO19 × CE/MAL/32	96.43	1087.69	592.06	64.91	3.24	8.12	1.05	79.97
KAO19 × CE/IND/16	110.13	2386.55	1248.34	67.97	4.06	8.89	0.91	83.42
BL/SM/10 × KAO19	114.31	1193.19	653.75	59.47	2.76	9.01	0.99	77.7
BL/SM/10× BL/SM/115	160.26	936.16	548.21	59.18	3.09	10.25	1.04	78.39
BL/SM/10× CE/MAL/32	166.64	546.91	356.78	56.93	3.06	9.77	0.89	76.14
BL/SM/10× CE/IND/16	121.10	1269.64	695.37	62.57	2.13	11.01	0.98	78.99
BL/SM/115× KAO19	199.84	888.29	544.06	67.86	2.46	11.30	0.97	78.53
BL/SM/115× BL/SM/10	75.93	1442.98	759.46	60.68	2.25	9.37	1.09	77.41
BL/SM/115×CE/MAL/32	96.88	484.11	290.49	57.78	2.38	7.77	1.1	74.12
BL/SM/115× CE/IND/16	250.35	975.28	612.81	68.48	2.89	9.185	1.08	73.53
CE/MAL/32× KAO19	56.92	705.62	381.27	55.23	2.17	7.99	0.94	83.08
CE/MAL/32× BL/SM/10	100.08	646.35	373.22	62.84	1.94	10.78	0.95	81.59
CE/MAL/32×BL/SM/115	72.63	700.45	386.54	59.99	2.16	9.28	0.79	78.11
CE/MAL/32× CE/IND/16	94.77	996.73	545.75	59.62	2.83	9.21	0.98	74.71
CE/IND/16× KAO19	83.04	801.03	442.03	56.47	2.99	9.97	0.97	80.05
CE/IND/16× BL/SM/10	258.73	1656.55	957.64	74.49	3.24	11.48	1.02	82.58
CE/IND/16× BL/SM/115	177.65	1591.3	1681.47	67.29	1.88	10.12	0.94	82.84
CE/IND/16× CE/MAL/32	168.21	2382.71	2031.46	68.56	2.63	11.29	0.97	77.49
Mean	131.42	1095.21	40.13	62.22	2.88	10.06	0.99	78.73
Lsd (p<0.05)	5.32	81.54	11.47	0.99	0.39	2.32	NS	4.96
Cv (%)	2.50	4.59	17.64	0.9855	8.27	14.22	47.99	3.89

PT = Phytate content(mg/100g); OX = Oxalate content(mg/100g); TN = Tannins content(mg/100g); MC = percentage moisture content; PTT = percentage Protein content; %TC = percentage carbohydrate; ASH = percentage Ash content; FAT = percentage fat content

Among the parents, CE/MAL/32 recorded the least (37.16 mg/100g) phytate content while KAO19 recorded the highest (268.48 mg/100g) phytate content. The oxalate content ranged from 563.58 mg/100g for BL/SM/115 to 1701.47 mg/100g for BL/SM/10. KAO 19 and CE/IND/16 also presented high oxalate content of 1409.21 mg/100g and 1347.63 mg/100g respectively. BL/SM/10 presented tannin content of 887.80 mg/100g among the parents while CE/MAL/32 had the least of 301.94 mg/100g (Table 4).

KAO 19 contained the highest amount of moisture of 67.91% while BL/SM/115 contained the least amount of 53.97%. The highest percentage ash was recorded by BL/SM/115 and the least recorded by BL/SM/10. The protein content among the parents ranged from 8.25% for KAO 19 to 14.65% for CE/MAL/32. Genotype BL/SM/10 was observed to have the highest fat content of 1.86 while CE/MAL/32 presented the least fat content of 0.75. Total carbohydrate was highest (82.37%) for CE/IND/16 and least (79.33%) for BL/SM/115 among the parent (Table 4).

For the F1 hybrids, progenies from KAO 19 × CE/IND/16 were observed to contain the highest oxalate content of 2386.55 mg/100g followed by progenies from CE/IND/16 × CE/MAL/32 while progenies from KAO19 × BL/SM/115 contained the least oxalate content of 372.89 mg/100g. Hybrids of CE/IND/16 × CE/MAL/32 presented the highest tannin content among the hybrids while KAO 19 × BL/SM/115 presented the least tannin content.Progenies from CE/MAL/32 × KAO 19 recorded the least (56.92 mg/100g) phytate content while progenies from CE/IND/16×BL/SM/10 recorded the highest (258.73 mg/100g) phytate content (Table 4).

Moisture content among the hybrids ranged from 55.23% for hybrids of CE/MAL/32 × KAO 19 to 74.49% for hybrids of CE/IND/16 × BL/SM/10. Progenies from KAO 19 × CE/IND/16 were observed to have the highest ash while progenies from CE/IND/16 × BL/SM/115 presented the least ash content. The protein content for the hybrids ranged from 7.77% to 12.17% for hybrids of BL/SM/115 × CE/MAL/32 and KAO 19 × BL/SM/115 respectively. The highest amount of carbohydrate (83.42%) was contained in hybrids KAO 19 × CE/IND/16 while the least (73.53%) was observed in hybrid BL/SM/115× CE/IND/16 (Table 4).

### 4.2 Environmental and genotypic effects on the nutrient and anti-nutrient composition

The environmental variance analysis showed highly significant (p<0.01) difference between the environments for all the nutrient and anti-nutrient composition studied (Table 5). The genotype by environment interaction (GEI) was also significant (p<0.01) for all the traits studied except for percentage fat content. The analysis also revealed that the magnitude of the mean square for all the traits was higher for the environment than the GEI and the genotype (Table 5).

**Table 5:** Mean square for nutrient and anti-nutrient composition of taro genotypes across three environments.

Source	df	PT	OX	TN	MC	ASH	PTT	FAT	TC
Env	1	26506.11***	5933568.91***	2044.19**	928.42***	35.37***	3069.26***	12.38***	3428.31***
Rep × Env	4	21.35	6893.33 *	137.84 *	0.47	0.01	0.13	0.173	8.41
Gen	24	26434.44***	1789569.06***	1190.38***	157.95***	2.43***	16.29 ***	0.25	49.29 ***
Env × Gen	24	14532.08***	1694247. 86***	998.93***	174.12v	2.51***	11.19 ***	0.28	45.09 ***
Error	96	10.76	2531.41	50.09	0.38	0.06	2.05	0.22	9.37
Total	146								

PT = Phytate content(mg/100g) OX = Oxalate content(mg/100g), TN = Tannins content(mg/100g), MC = percentage moisture content, PTT = percentage Protein content %TC = percentage carbohydrate, ASH = percentage Ash content and FAT = percentage fat content.

*, ** and *** = Significance at p<0.05, P<0.01 and p<0.001 respectively.

The environmental means for the nutrient and anti-nutrient composition of the parents and hybrids are presented in Table 6. The analysis revealed that the means of the anti-nutrient composition (Oxalate, Tannin and Phytate) were lower in environment 1 than environment 2. For the nutritional composition; percentage ash, percentage protein and percentage fat contents were higher in environment 1 while moisture content and total carbohydrate were also higher in environment 2.

**Table 6:** Environmental mean for the nutrient and anti-nutrient composition of taro genotypes.

Trait	Bunso	Tano Dumasi
PT	118.13	144.72
OX	896.32	1294.10
TN	36.44	43.82
MC	59.73	64.71
ASH	3.37	2.39
PTT	14.59	5.54
FAT	1.27	0.70
TC	73.95	83.51

PT = Phytate content(mg/100g) OX = Oxalate content(mg/100g)

TN = Tannins content(mg/100g), MC = percentage moisture content

PTT = percentage Protein content %TC = percentage carbohydrate

ASH = percentage Ash content and FAT = percentage fat content.

The AMMI analysis of variance revealed highly significant (p<0.001) genotype, environment and GEI effects for all the nutrient and anti-nutrient compositions for the parents and hybrids evaluated (Table 7).

**Table 7:** AMMI analysis of variance showing sum of squares and its significance test and % total variation of environment, genotype and GEI for nutrient and anti-nutrient compositions among taro genotypes across two environments.

		Ash		PTT		TC		MC	
Source	df	F test SS	TV (%)	F test SS	TV (%)	F test SS	TV (%)	F test SS	TV (%)
Total	149	159.32		3929		6627		8936	
Treatments	49	153.85***		3729***		5694		8898***	
Genotypes	24	58.30***	36.59%	391***	9.95%	1183	17.85%	3791***	42.42%
Environments	1	35.36***	22.19%	3069***	78.11%	3428	66.27%	928***	10.38%
BLOCK	4	0.02		1		34		2	
GEI	24	60.19***	37.78%	269***	6.85%	1083	16.34%	4179***	46.76%
Residuals	22	0.00		0.00		0.00		0.00	
Error	96	5.44		197					
		Fat		OX		PT		TN	
Source	df	F test SS	TV (%)	F test SS	TV (%)	F test SS	TV (%)	F test SS	TV (%)
Total	149	47.29		89815672		1010820		59946	
Treatments	49	25.17***		89545090***		10097802***		54586***	
Genotypes	24	5.96***	12.60%	42949545***	47.82%	634425***	62.76%	28568***	47.65%
Environments	1	12.36***	26.15%	5933533***	6.61%	26507***	2.62%	2044***	3.41%
BLOCK	4	0.70		27571		85		551	
GEI	24	6.83***	14.44%	40662012***	45.27%	348770***	34.50%	23974***	39.99%
Residuals	22	0.00		0.00		0.00		0.00	
Error	96	21.42		243011		1033		4809	

Only one IPCA accounted for the total GEI for all the traits. The GEI effect was the highest contributor to the total variation for ash content (37.78%) and moisture content (46.76%). Environmental effect was the highest contributor to the total variation for protein content (78.11%), total carbohydrate (66.27%) and fat content (26.15%).Genotypic effect was the highest contributor to the total variation for oxalate, phytate and tannin content (Table 7).

### 4.3 Phenotypic plasticity of the parents and hybrids of taro for nutrient and anti-nutrient composition

**Fig 1:**
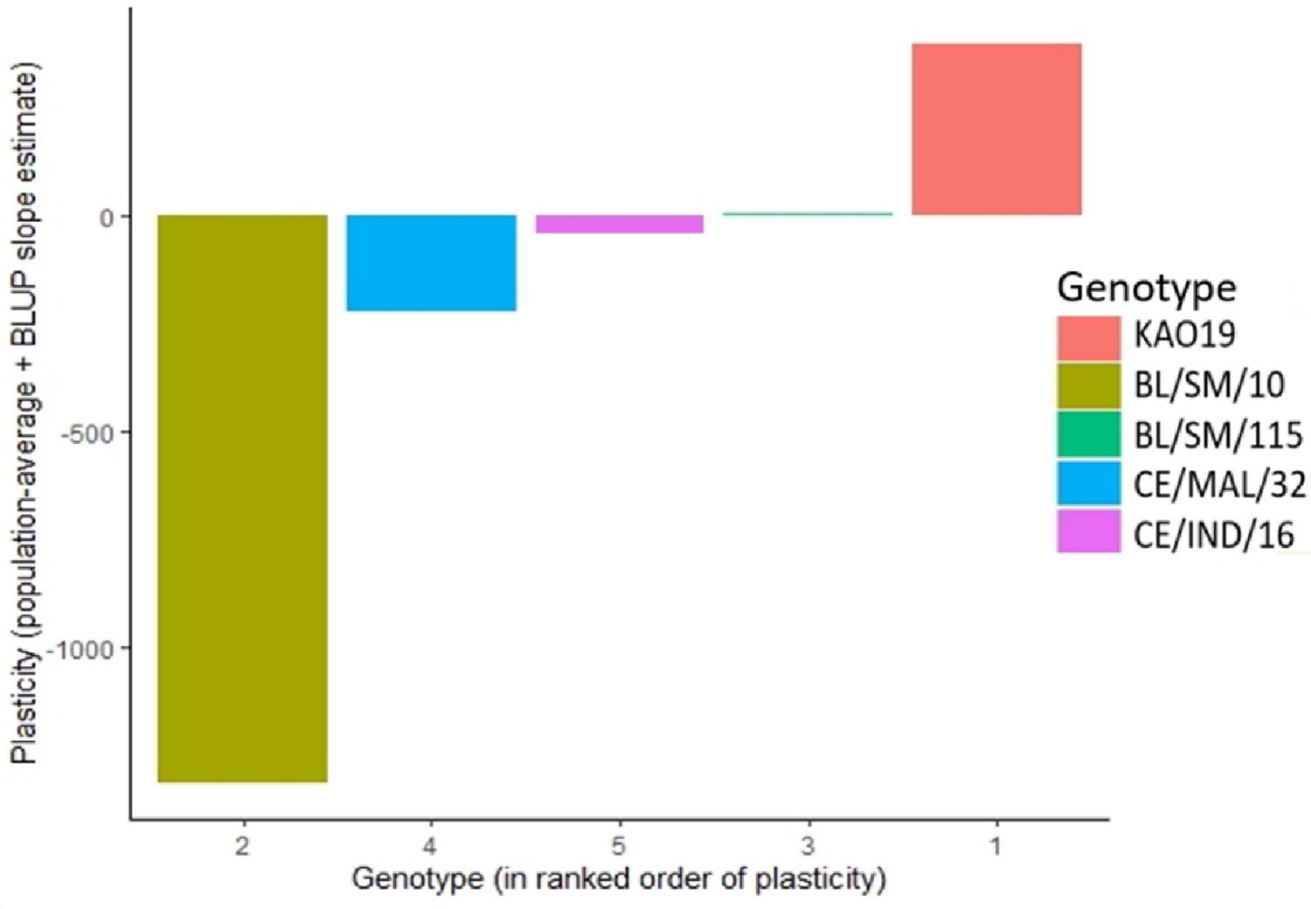
Plasticity of parents for the combined nutrient and anti-nutrient composition.

**Fig 2:**
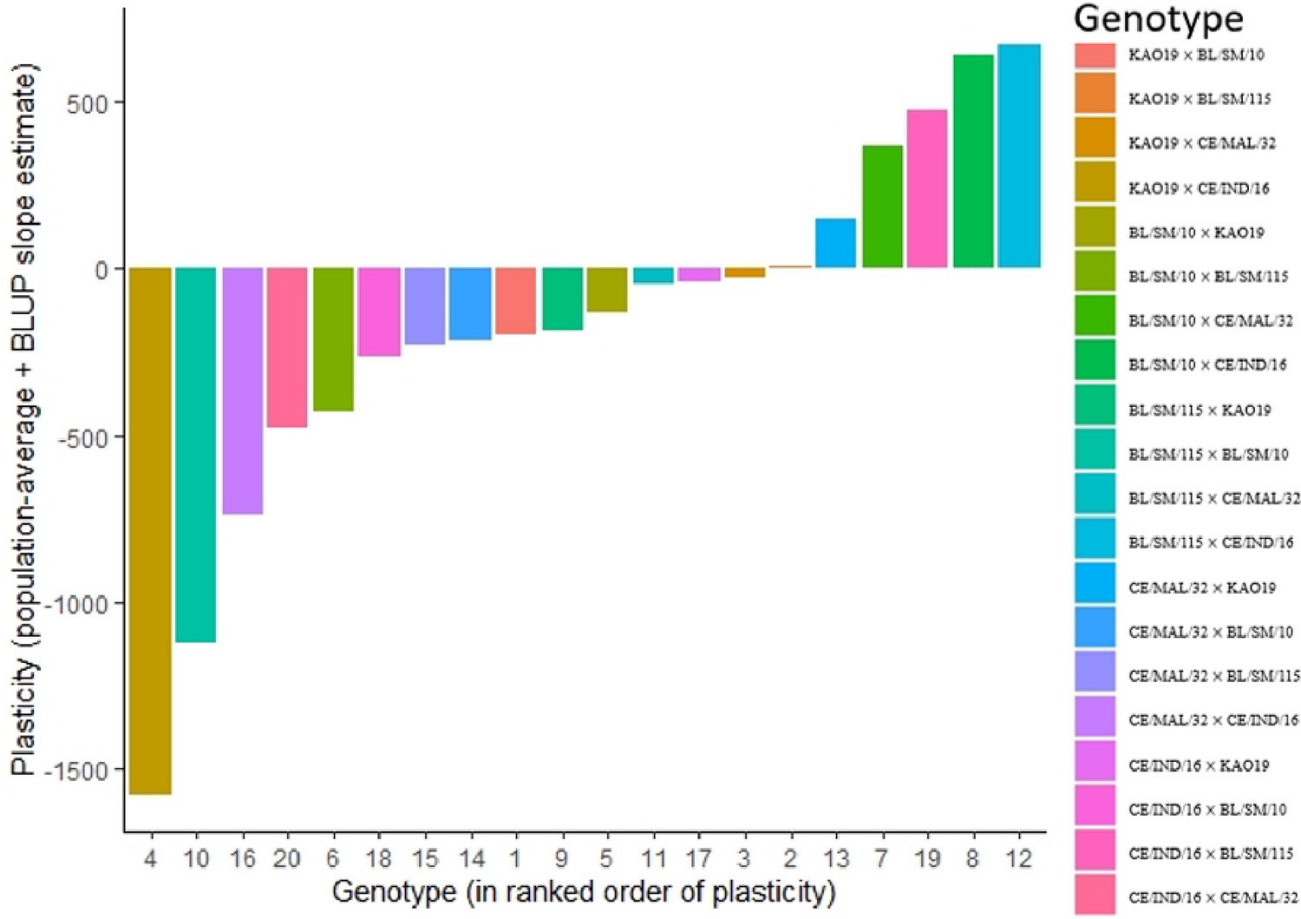
Plasticity of hybrids for the combined nutrient and anti-nutrient composition.

The phenotypic plasticity index (PPI) (Figs 1 and 2) revealed stability for both parents and hybrids for the nutrient and anti- nutrient composition. Genotypes which recorded near zero PPI are more stable while those that recorded above zero PPI are more plastic.Parents BL/SM/10, CE/MAL/32 and CE/IND/16 were observed to be stable for combined nutrient and anti-nutrient composition studied as they recorded near zero PPI. KAO19 was however more plastic, recording above zero PP1 (Fig 1). For the hybrids, KAO19 x CE/IND/16 was observed to be highly stable for the combined nutrient and anti-nutrient composition followed by BL/SM/115 × BL/SM/10, CE/MAL/32 × CE/IND/16 and CE/IND/16 × CE/MAL/32 respectively. Hybrid BL/SM/115 × CE/IND/16 was however observed to be highly plastic for the combines nutrient and anti-nutrient composition (Fig 2).

The nutrient composition showed variable degree of stability (Tables 8 and 9). The average PPI for the traits ranged from 0.23 for oxalate content to 1.14 for tannin content.High plasticity was observed for the parents than the hybrids for the nutrient composition except for ash, oxalate and tannins contents with an average PPI of 0.60, 0.11 and 0.88 respectively for parents and 0.794, 0.29 and 1.23 respectively for hybrids.

**Table 8:** Phenotypic plasticity index (PPI) for nutrient and anti-nutrient composition in parental entries.

Parents	Ash	Protein	CHO	Fat	MC	Oxalate	Phytate	Tannins
KA 019	0.22	0.94	**1.54**	0.49	0.49	0.17	0.71	0.26
BL/SM/10	0.1	**3.08**	**1.06**	**1.5**	0.28	0.31	0.45	0.71
BL/SM/115	**1.14**	0.6	0.44	0.89	0.39	0.00	0.63	0.33
CE/MAL/32	**1.2**	**1.61**	0.84	0.35	0.79	0.07	**1.14**	**1.34**
CE/IND/16	0.34	**1.5**	**1.94**	0.32	**1.27**	0.01	**0.17**	**1.77**
**Average**	0.60	1.55	1.16	0.71	0.64	0.11	0.62	0.88

**Table 9:** Phenotypic plasticity index (PPI) for nutrient and anti-nutrient composition in hybrids of taro.

Hybrids	Ash	Protein	CHO	Fat	MC	Oxalate	Phytate	Tannins
KAO19 × BL/SM/10	0.59	0.37	0.16	0.28	0.39	0.08	0.64	0.26
KAO19 × BL/SM/115	0.53	0.61	0.93	0.69	0.94	0.00	0.89	**1.10**
KAO19 × CE/MAL/32	**2.41**	**1.08**	**2.21**	0.53	**1.51**	0.01	**1.42**	**1.75**
KAO19 × CE/IND/16	0.02	0.39	**1.98**	0.67	0.31	0.53	0.66	0.26
BL/SM/10 × KAO19	**1.11**	**1.13**	0.51	0.45	0.56	0.07	0.15	0.68
BL/SM/10× BL/SM/115	0.96	**2.08**	0.47	0.69	0.19	0.50	0.96	0.42
BL/SM/10× CE/MAL/32	0.47	0.99	0.37	0.30	0.28	0.27	0.79	0.60
BL/SM/10× CE/IND/16	0.22	**1.00**	0.36	0.54	0.39	0.68	0.22	**2.34**
BL/SM/115× KAO19	0.01	0.66	0.85	0.46	0.29	0.02	**1.15**	0.27
BL/SM/115× BL/SM/10	0.05	**1.21**	0.41	0.50	0.97	0.23	0.62	0.45
BL/SM/115× CE/MAL/32	**1.10**	0.55	0.30	0.59	0.91	0.00	0.27	0.47
BL/SM/115× CE/IND/16	0.19	**1.29**	0.34	**1.03**	0.28	0.72	0.54	**2.21**
CE/MAL/32× KAO19	0.40	0.79	**1.24**	0.34	0.53	0.04	0.19	**2.09**
CE/MAL/32× BL/SM/10	0.01	0.68	0.45	0.65	0.21	0.08	0.37	**1.70**
CE/MAL/32× BL/SM/115	0.35	0.90	**1.41**	0.35	0.92	0.09	0.77	**1.75**
CE/MAL/32× CE/IND/16	**1.44**	0.59	0.13	0.11	0.62	**1.03**	0.42	0.63
CE/IND/16× KAO19	0.69	0.72	**1.30**	0.35	0.20	0.00	0.21	**2.47**
CE/IND/16× BL/SM/10	0.79	**1.63**	**2.18**	0.14	**1.49**	0.20	0.41	**2.25**
CE/IND/16× BL/SM/115	**4.53**	**1.58**	0.32	0.27	0.44	0.19	0.45	0.54
CE/IND/16× CE/MAL/32	0.01	**1.59**	**1.48**	0.04	**1.10**	0.63	0.77	**2.11**
**Average**	0.79	0.99	0.87	0.45	0.63	0.27	0.59	1.23

PT = Phytate content(mg/100g); OX = Oxalate content(mg/100g); TN = Tannins content(mg/100g); MC = percentage moisture content; PTT = percentage Protein content; %TC = percentage carbohydrate; ASH = percentage Ash content;FAT = percentage fat content. PPI above 1.00 are bold.

The average PPI for the parents ranged from 0.11 for oxalate content to 1.55 for protein content. Among the parents, BL/SM/10 was stable with PPI of 0.1 while CE/MAL/32 was plastic with PPI of 1.2 for ash content. BL/SM/10 recorded the highest PPI of 3.08 while BL/SM/115 had the least PPI of 0.6. For total carbohydrates, BL/SM/115 was more stable while CE/IND/16 was more plastic. The PPI for fat content ranged from 0.32 for CE/IND/16 to 1.5 for BL/SM/10. All the parents recorded less than one PPI for moisture content except for BL/SM/10 which had PPI of 1.27. All the parents were more stable for oxalate contents as they recorded PPI of less than one with BL/SM/115 recording PPI of 0.00. For phytate content, CE/IND/16 was more stable with PPI of 0.17 while CE/MAL/32 was more plastic with PPI of 1.14. CE/MAL/32and CE/IND/16 were more plastic for tannin content as they recorded PPI of more than 1 while KA 019, BL/SM/10 and BL/SM/115 were more stable (Table 8).

The nutrient and anti-nutrient composition of the hybrids showed variable stability with tannins recording the highest average PPI of 1.15 and fat content recording the least of 0.45.Progenies from BL/SM/115 × CE/MAL/32, CE/MAL/32 × CINDY/16, CE/IND/16 × CE/MAL/32 were more plastic for ash content with above 1.00 PPI. The PPI for protein content ranged from 0.37 for KA 019 X BL/SM/10 to 2.08 for BL/SM/10 × BL/SM/115. Progenies from KA 019 × CE/MAL/32, KA 019 × CE/IND/16, CE/MAL/32 × KA 019, CE/MAL/32 × BL/SM/115, CE/IND/16 × KA 019, CE/IND/16 × BL/SM/10 and CE/IND/16 × CE/MAL/32 showed more plasticity for total carbohydrates, CE/MAL/32 × CE/IND/16was most stable with PPI of 0.13 for total carbohydrate. All the hybrids for the crosses were stable for fat content except for the hybrids of BL/SM/115 × CE/IND/16 which had PP1 of 1.03. The average PPI for moisture content was 0.63 with progenies from KA 019 × CE/MAL/32, CE/IND/16× BL/SM/10 and CE/IND/16× CE/MAL/32 being more plastic with PP1 of above 1.0. All the hybrids of the crosses recorded PPI of less than 1.0 except for the hybrid of CE/MAL/32 × CE/IND/16for oxalate content with progenies from KA 019 × BL/SM/115, BL/SM/115 × KA 019, BL/SM/115 × CE/MAL/32, CE/MAL/32 × KA 019 and CE/IND/16 × KA 019 having PPI of less than 0.1. The PPI for phytate content among the hybrids ranged from 0.19 for progenies from CE/MAL/32 × KA 019 to 1.42 for progenies from KA 019 × CE/MAL/32. Hybrids of the crosses KA 019 × BL/SM/115, KA 019 × CE/MAL/32, BL/SM/10 × CE/MAL/32, BL/SM/115 × CE/IND/16, CE/MAL/32 × KA 019, CE/MAL/32 × BL/SM/10, CE/MAL/32 × BL/SM/115, CE/IND/16 × BL/SM/10, CE/MAL/32× KA 019 and CE/IND/16 × CE/MAL/32were more plastic for tannin content as they presented PPI of greater than 1.0, however, hybrids of crosses KA 019× BL/SM/10, KA 019× CE/IND/16, BL/SM/115× KA 019were more stable (Table 9).

### 4.5 Association among nutrient and anti-nutrient composition for taro genotypes

The correlation analysis for nutrient and anti-nutrient composition is presented in Table 10. The analysis revealed positive and highly significant (p<0.001) correlation between phytate and oxalate content (r = 0.49). Tannin (r = 0.89) and moisture (r = 0.68) contents showed positive and highly significant (p<0.001) correlation with oxalate contents. Percentage ash was negatively correlated with all the traits studied except for protein content (r = 0.33) although not significant (p>0.05). There was non-significant (p>0.05) negative correlation between protein content and oxalate content (r = -0.05) and non-significant positive correlation between phytate (r = 0.09) content.Total carbohydrate was also positively correlated with phytate (r = 0.14), oxalate (r = 0.42) and tannin (r = 0.34) contents although not significant (p>0.05). Fat content was also positively associated with oxalate (r = 0.28) and tannin (r = 0.34) content while negatively associated with phytate (r = -0.05) content (Table 10).

**Table 10:** Correlation matrix for nutrient and anti-nutrient composition in taro.

	PT	OX	TN	MC	ASH	PTT	FAT
OX	0.49**						
TN	0.83***	0.89***					
MC	0.42	0.68***	0.65***				
ASH	-0.25	0.78	-0.17	-0.08			
PTT	0.09	-0.05	0.002	0.01	0.33		
FAT	-0.05	0.28	0.15	0.19	-0.40	-0.19	
TC	0.14	0.42	0.34	0.33	-0.08	0.02	-0.09

PT = Phytate content (mg/100g); OX = Oxalate content (mg/100g); TN = Tannins content (mg/100g); MC = percentage moisture content; PTT = percentage Protein content; %TC = percentage carbohydrate; ASH = percentage Ash content; FAT = percentage fat content.

*, ** and *** = Significance at p<0.05, P<0.01 and p<0.001 respectively.

## 5.0 Discussion

### 5.1 Diversity among the genotypes for nutritional and anti- nutrient compositions

Developing taro genotypes with enhanced nutritional and low anti-nutritional composition is important for improved human nutrition and industrial purposes. Assessing the nutritional profile of accessions provides important information for crop improvements [31, 32] (Gurmu *et al*., 2014: Khatemenla *et al*., 2019). Genotypes which contain less amount of anti-nutrients, high amount of nutrient and low moisture content are preferable by consumers since they are seem to be of high quality [32] (Khatemenla *et al*., 2019). The significant differences among the accessions for the nutritional and anti-nutritional traits indicates genetic variability among the genotypes and also suggests that, good selection responsecan be made in the development of taro varieties with high nutritional and low anti-nutrient compositions [32–34]. Boampong *et al*. (2019), Khatemenla *et al*., (2019) and Matikiti *et al*., 2017 reported diversity among taro accessions for nutritional traits [6]. Angami *et al*. (2015) and [35] Azene and Molla (2017) have also reported similar results on oxalic and moisture content in taro. The finding of the research also agrees with the works of [36] Gurmu *et al*. (2020) and [19] Tumwegamire *et al*. (2016) who reported significant differences among newly developed sweet potato clones for nutritional composition [37]. Polycarp *et al*. (2012) also reported significant difference in chemical factors and anti-nutritional composition in Ghanaian yams.

Breeding for high nutritional composition such as high protein, ash and carbohydrate as well as low fat, oxalate, phytate, tannin and moisture contents in crops is important for improving the health of humans ([36, 38] Magwaza *et al*., 2016; Gurmu *et al*., 2020). Therefore, selecting of taro genotypes with high protein, ash and carbohydrate contents and low fat, oxalate, phytate, tannin and moisture contents will serve as useful genetic resource for improving human nutrition in Ghana. Hybrids from KAO 19 × CE/IND/16, CE/MAL/32 × KAO19, KAO 19 × BL/SM/115 and CE/IND/16 × BL/SM/115 and genotypes BL/SM/115, CE/MAL/32 and BL/SM/10 which recorded high protein, ash and carbohydrate content with low fat, oxalate, phytate, tannin and moisture contents below the daily recommended amount for human consumption ([39, 40] Massey *et al*., 2001; Reddy *et al*., 1982) can be included in breeding programs for the nutritional improvement of taro in Ghana.

### 5.2 Environmental and genotypic effects of the genotypes for nutritional and anti- nutrient compositions

The significant differences between the environments indicate the diversity among the environments used for the research and this is evidence in the differences in the climatic conditions that existed in the two agro-ecological zones used for the research (Table 2).The higher magnitude of the environment over genotype and GEI indicates the influence of environment in the expression of the nutritional and anti-nutritional traits making environment an important factor in the breeding of taro genotypes for nutrient and anti-nutrients composition. The higher levels of the nutritional composition and the lower levels of the anti-nutritional composition among the genotypes cultivated at Bunso, suggests Bunso to be an ideal environment for cultivation of taro for improved nutritional composition. The higher levels of the anti-nutritional composition of the genotypes at the Tano Dumasi environment may be due to high soil moisture content as Tano Dumasi research site was a waterlogged area and acridity content of taro increases when it comes into contact with moisture [41] (Fufa *et al*., 2021). These findings are in agreement with the work of [42] Huang *et al*. (2007) who reported higher anti-nutrient composition among taro genotypes in paddy areas than in upland fields.

The higher genotypic effect on oxalate, phytate and tannin composition suggests the presence of high level of diversity among the genotypes for anti-nutrient traits studied. This gives opportunity for selecting superior genotypes among the genotypes used for the research for further evaluation and onward distribution to farmers for cultivation or to include them in breeding programs for varietal development of taro for low anti-nutrient content in Ghana [43]. Nduwumuremyi (2017) also reported high genotypic effects on carotene content in cassava. [44] Oduro (2013) and [45] Gurmu (2015) also reported high genotype effect on nutritional traits in potatoes [46]. Brown *et al*. (2010) however, reported non-significant (p>0.05) environment and genotype effect on mineral content in potatoes.

The higher environment effect on protein, carbohydrate and fat contents indicate that the variability among the genotypes for those traits are largely influenced by environment. Therefore, specific selection for protein, carbohydrate and fat is recommended to overcome environmental influence [36]. Gurmu *et al*. (2020) [19], Tumwegamire *et al*. (2016) and [47] Haynes *et al*. (2010) have reported similar results for nutritional content in potatoes.

### 5.3 Phenotypic plasticity and stability of the genotypes for nutritional and anti- nutrient composition

The varied phenotypic plasticity among the genotypes for the nutrient and anti-nutrients composition is an indication of varied adaptation of the genotypes to the environment. It also confirms the effect of GEI in the expression of the traits [48]. (Des Marais *et al*., 2013). Genotypes that exhibited PPI of near zero for most of the nutrient and anti-nutrient compositions are more stable across the environments [49] (Tan *et al*., 2020).These genotypes can therefore be recommended for cultivation in wide range of environments. Therefore, genotypes KAO19 and BL/SM/115 × CE/IND/16 can be recommended for cultivation in a wide range of environments for cormquality. The genotypes which presented high PPI are more plastic and unstable and therefore can be selected for particular environments. Phenotypic plasticity among taro genotypes for nutritional traits has not been reported in literature, however, [22] Hidayatullah *et al*. (2020) reported phenotypic plasticity for eddoe and dasheen taro for yield and yield components [23]. Tripodi *et al*. (2020) reported plasticity among chilli pepper genotypes for health-related compounds in two environments [49]. Tan *et al*. (2020) also reported on phenotypic plasticity of the rice grain ionome.

### 5.4 Association among the nutritional and anti-nutritional compositions

Significant and positive correlation among traits indicates that the traits can be improved together while significant and negative correlations indicate that the traits will have to be improved separately.The inter-correlation between all the anti-nutrients indicates that they can be improved together. The negative correlation between the anti-nutrients studied and protein and ash contents which are important nutritional qualities suggest that these traits can be improved together since low values of the anti-nutrients in the taro cormis desirable [50]. Mwenye *et al*. (2011) also observed positive and negative significant correlations among nutritional composition of taro [51]. Ouédraogo *et al*. (2023) also observed positive correlation between nutritional compositions in taro genotypes in Burkina Faso.

## 6.0 Conclusions

There were lots of variability among the genotypes of taro studied for nutritional and anti-nutritional compositions. Environment played an important role in the expression of the nutrients and anti- nutrients compositions as the genotypes showed varied levels of stability for the traits. Genotypes BL/SM/115, CE/MAL/32 and CE/IND/16 which recorded high nutrient and low anti-nutrient contents and were stable across the environments can be included in breeding programs for the development of enhanced nutritional quality taro varieties. Crosses KAO19 × CE/MAL/32 and CE/IND/16×KAO19, CE/IND/16 × BL/SM/10, CE/IND/16 × BL/SM/115 which also showed stability with high nutrients and low anti-nutrients can be released to farmers for cultivation.

## Supporting information

S1 File Mean of taro genotypes for nutrients and anti-nutrients compositions in environment 1 and 2. (PDF)

S1 Data Evaluation and plasticity data. (XLSX)
