# Supplementary material for: Evaluation and phenotypic plasticity of taro [Colocasia esculenta (l.) Schott.] genotypes for nutrient and anti-nutrient composition
Source: PLoS One. 2023 Sep 13;18(9):e0291358. doi: 10.1371/journal.pone.0291358 (PMC10499218; doi:10.1371/journal.pone.0291358)
Supplement: S1 File — (PDF) [file pone.0291358.s001.pdf]

S1Table 1: Mean of taro genotypes for nutrients and anti-nutrients compositions in environment 1

Genotypes	Ash	PTT	TC	Fat	MC	Oxalate	PT	TN
Gen01	3.07	12.77	72.11	1.29	63.00	1115.00	205.20	40.63
Gen02	2.11	17.84	74.22	1.19	64.57	2938.50	124.67	59.54
Gen03	2.96	18.26	71.58	1.32	49.53	553.10	71.20	30.20
Gen04	2.81	17.84	74.25	1.04	59.74	755.50	62.57	11.21
Gen05	3.53	17.21	74.25	1.07	60.02	1379.30	139.17	44.31
Gen06	3.58	10.66	74.62	1.20	62.35	921.30	194.26	46.63
Gen07	4.05	15.77	71.52	1.30	62.94	387.30	166.09	23.73
Gen08	4.70	12.90	71.00	1.42	62.28	1095.30	104.52	76.63
Gen09	4.11	11.74	75.12	1.33	68.62	4013.60	48.97	43.72
Gen10	3.74	13.94	73.79	1.34	54.19	1361.10	85.41	27.76
Gen11	4.01	16.94	77.25	1.45	58.27	1437.40	86.63	32.75
Gen12	3.69	14.37	74.11	1.16	53.22	180.60	160.37	26.50
Gen13	2.57	15.58	78.30	1.35	61.51	688.70	85.88	10.80
Gen14	2.44	13.72	73.25	1.31	57.98	998.70	280.44	62.09
Gen15	2.46	14.13	73.80	1.42	67.64	2524.60	94.59	52.64
Gen16	3.37	9.43	72.95	1.49	57.51	530.60	109.19	31.24
Gen17	3.30	14.48	71.72	1.59	56.69	376.50	195.50	32.37
Gen18	2.77	12.01	76.39	1.23	53.69	558.50	60.23	26.11
Gen19	1.94	14.40	79.22	1.35	61.95	840.20	114.48	46.71
Gen20	2.71	13.69	73.54	1.08	53.23	922.50	93.25	11.52
Gen21	3.96	12.75	73.00	1.15	60.20	1716.00	90.07	46.23
Gen22	3.77	13.94	73.69	1.27	59.63	858.50	93.94	17.00
Gen23	4.05	17.43	73.80	1.19	65.89	1975.40	60.19	36.19
Gen24	5.89	16.00	73.59	1.19	52.50	1285.60	112.20	33.20
Gen25	2.56	16.85	71.88	1.06	61.14	2938.50	114.26	41.19
Grand Mean	3.37	14.59	73.95	1.27	59.73	1294.10	118.13	36.44
CV	9.30	13.40	1.90	4.9	1.10	4.60	3.20	17.70
LSD	0.51	3.21	2.29	0.10	1.12	98.62	6.17	10.57
P value	<.001	<.001	<.001	<.001	<.001	<.001	<.001	<.001

S1 Table 2: Mean of taro genotypes for nutrients and anti-nutrients compositions in environment 2

Genotypes	Ash	PTT	TC	Fat	MC	Oxalate	PT	TN
Gen01	2.33	3.74	87.19	0.58	72.82	1703.40	331.76	98.47
Gen02	1.67	2.72	84.45	2.51	63.59	464.40	23.59	55.36
Gen03	4.77	11.03	79.17	0.36	58.42	574.00	59.31	27.42
Gen04	4.88	6.03	85.38	0.45	55.99	377.90	11.75	7.30
Gen05	2.49	6.25	90.82	0.48	64.94	1316.00	76.94	81.63
Gen06	2.16	4.94	78.55	0.69	53.58	533.20	204.94	42.89
Gen07	3.35	8.56	81.24	0.47	58.82	358.40	180.02	61.96
Gen08	1.78	3.34	88.95	0.68	67.55	1080.10	88.33	46.62
Gen09	4.00	6.05	91.71	0.49	67.32	759.50	171.29	25.04
Gen10	1.78	4.07	81.61	0.65	64.75	1025.20	143.22	35.95
Gen11	2.16	3.56	79.54	0.62	60.09	435.00	233.88	47.76
Gen12	2.43	5.18	78.16	0.61	60.65	913.20	172.91	17.92
Gen13	1.70	6.43	79.69	0.61	63.63	1850.60	156.33	66.27
Gen14	2.48	8.88	83.80	0.63	77.75	777.90	119.24	45.91
Gen15	2.05	4.61	81.02	0.76	53.73	361.40	57.26	30.22
Gen16	1.39	6.11	75.29	0.71	58.04	437.60	84.56	55.50
Gen17	2.46	3.89	75.35	0.57	80.26	1574.00	305.20	48.38
Gen18	1.57	3.96	89.77	0.65	56.77	852.70	53.61	30.17
Gen19	1.94	7.16	83.98	0.55	63.74	452.50	85.68	32.87
Gen20	1.60	4.86	82.68	0.49	66.76	478.40	52.01	26.02
Gen21	1.69	5.66	76.40	0.81	54.03	277.40	99.47	17.80
Gen22	2.24	6.00	86.40	0.67	53.29	743.50	72.13	74.58
Gen23	2.42	5.52	91.35	0.83	83.09	1337.70	157.26	32.52
Gen24	1.88	4.23	92.08	0.67	82.09	1897.00	431.10	30.66
Gen25	2.63	5.73	83.09	0.88	75.98	1826.90	246.15	56.25
Grand Mean	2.39	5.54	83.51	0.69	64.71	896.30	144.72	43.82
CV	5.30	9.30	4.90	16.80	0.80	4.30	1.90	17.50
LSD	0.21	0.85	6.72	1.09	0.88	62.60	4.46	12.59
P value	<.001	<.001	<.001	0.41	<.001	<.001	<.001	<.001

PT= Phytate content(mg/100g) OX = Oxalate content(mg/100g), TN = Tannins content(mg/100g), MC = percentage moisture content, PTT = percentage Protein content %TC= percentage carbohydrate, ASH = percentage Ash content and FAT = percentage fat content
